# A comparison of heat-stress transcriptome changes between wild-type *Arabidopsis* pollen and a heat-sensitive mutant harboring a knockout of *cyclic nucleotide-gated cation channel 16* (*cngc16*)

**DOI:** 10.1186/s12864-018-4930-4

**Published:** 2018-07-24

**Authors:** Maryam Rahmati Ishka, Elizabeth Brown, Chrystle Weigand, Richard L. Tillett, Karen A. Schlauch, Gad Miller, Jeffrey F. Harper

**Affiliations:** 1 Department of Biochemistry and Molecular Biology, University of Nevada, Reno, MS330, Howard Building, Reno, NV 89557 USA; 2 Nevada INBRE Bioinformatics Core, University of Nevada, Reno, Reno, NV 89557 USA; 3 The Mina and Everard Goodman Faculty of Life Sciences Bar Ilan University, 52900 Ramat-Gan, Israel

**Keywords:** *Arabidopsis thaliana*, Cyclic nucleotide-gated cation channel 16, Heat stress, Pollen, Transcriptome

## Abstract

**Background:**

In flowering plants, the male gametophyte (pollen) is one of the most vulnerable cells to temperature stress. In *Arabidopsis thaliana*, a pollen-specific *Cyclic* *Nucleotide-Gated cation* *Channel 16* (*cngc16*), is required for plant reproduction under temperature-stress conditions. Plants harboring a *cncg16* knockout are nearly sterile under conditions of hot days and cold nights. To understand the underlying cause, RNA-Seq was used to compare the pollen transcriptomes of wild type (WT) and *cngc16* under normal and heat stress (HS) conditions.

**Results:**

Here we show that a heat-stress response (HSR) in WT pollen resulted in 2102 statistically significant transcriptome changes (≥ 2-fold changes with adjusted *p*-value ≤0.01), representing approximately 15% of 14,226 quantified transcripts. Of these changes, 89 corresponded to transcription factors, with 27 showing a preferential expression in pollen over seedling tissues. In contrast to WT, *cngc16* pollen showed 1.9-fold more HS-dependent changes (3936 total, with 2776 differences between WT and *cngc16*). In a quantitative direct comparison between WT and *cngc16* transcriptomes, the number of statistically significant differences increased from 21 pre-existing differences under normal conditions to 192 differences under HS. Of the 20 HS-dependent changes in WT that were most different in *cngc16*, half corresponded to genes encoding proteins predicted to impact cell wall features or membrane dynamics.

**Conclusions:**

Results here define an extensive HS-dependent reprogramming of approximately 15% of the WT pollen transcriptome, and identify at least 27 transcription factor changes that could provide unique contributions to a pollen HSR. The number of statistically significant transcriptome differences between WT and *cngc16* increased by more than 9-fold under HS, with most of the largest magnitude changes having the potential to specifically impact cell walls or membrane dynamics, and thereby potentiate *cngc16* pollen to be hypersensitive to HS. However, HS-hypersensitivity could also be caused by the extensive number of differences throughout the transcriptome having a cumulative effect on multiple cellular pathways required for tip growth and fertilization. Regardless, results here support a model in which a functional HS-dependent reprogramming of the pollen transcriptome requires a specific calcium-permeable *Cyclic Nucleotide-Gated cation Channel, CNGC16.*

**Electronic supplementary material:**

The online version of this article (10.1186/s12864-018-4930-4) contains supplementary material, which is available to authorized users.

## Background

Temperature stress is a major contributor to crop loss around the world, with pollen infertility being one of the most important underlying causes [1–6]. Fertilization during plant reproduction is highly sensitive to hot and cold temperatures, with even a single hot day or cold night carrying the potential to disrupt reproductive success. Given the uncertainties of climate change, understanding this vulnerability is significant because most of the world’s food supply is derived from seed crops that depend on pollen fertilization.

Plant cells are proposed to sense heat stress (HS) through several mechanisms [7–10], including 1) accumulation of ROS (reactive oxygen species), 2) temperature-responsive calcium (Ca^2+^) channels, 3) an endoplasmic reticulum (ER)-unfolded protein response (UPR), 4) increased membrane fluidity, and 5) increased transcriptional activities related to a temperature-sensitive binding affinity of histones to specific regions of chromatin, leading to access of transcriptional regulators that can alter transcription.

Several transcriptome studies have been conducted to gain insights into heat stress responses (HSRs) in flowering plants during reproductive development, including both microarray and RNA-Seq experiments (reviewed in [11, 12]). However, only a few studies examined isolated mature pollen grains, and no pollen HS-studies have been reported for *Arabidopsis*. Nevertheless, studies in rice [13–15] and tomato [16–19] support an expectation that pollen and other reproductive tissues respond to HS with global changes in gene expression, including changes in the abundance of transcripts encoding small heat shock proteins (HSPs) [14–16, 18], heat shock transcription factors (HSFs*,* e.g. *HsfA2*) [14, 16, 18], enzymes involved in ROS mitigation (such as ascorbate peroxidase 1 (APX1) and catalase 2 (CAT2)) [14–16], proteins associated with the ER-unfolded protein response (such as HSPs, e.g. Hsp90 and CDC48, a homohexameric AAA-ATPase chaperone) [16, 18], and membrane trafficking (such as vesicle-associated membrane proteins AtVAMP725 and AtVAMP726) [16]. In addition, RNA-Seq has allowed the discovery of HS-dependent changes in small non-coding RNAs (sncRNAs) and alternative splicing events in tomato pollen [17, 18].

A previous study from Tunc-Ozdemir et al. [20] identified *Arabidopsis* *Cyclic* *Nucleotide-Gated cation* *Channel 16* (*CNGC16*) as a necessary component for pollen fertility under multiple stress conditions, including HS. A loss-of-function mutation of *cngc16* (i.e., knockout) resulted in a 10-fold reduction in pollen fitness and seed production under a HS condition. To obtain mechanistic insights into this hypersensitivity, we performed an RNA-Seq experiment to compare the pollen transcriptomes from a *cngc16* mutant and WT Col-0, with and without a temperature stress (hot/cold stress regime described in [20]). Results here define an extensive HS-dependent reprogramming of approximately 15% of the WT pollen transcriptome, and identify at least 27 transcription factor associated changes that could provide unique contributions to a pollen HSR. In contrast to WT, pollen from a *cngc16* mutant showed 1.9-fold more HS-dependent transcriptome changes. These results support a model in which HS-tolerance in pollen involves an extensive reprogramming of the transcriptome through a signaling pathway that is dependent on the function of a specific *Cyclic Nucleotide-Gated cation Channel, CNGC16.*

## Results

To compare *cngc16* and WT pollen for differences in their response to a temperature stress condition, transcriptomes were analyzed from mature pollen grains harvested at midday from plants grown under control (normal) conditions or a HS regime. For the stress condition, plants were grown under a diurnal cycle of hot and cold temperatures (Additional file 1) [20] for a period of 1 week, and pollen were harvested at the end of the HS period that peaked at 40 °C. Thus, many of the harvested pollen grains matured under multiple cycles of hot and cold stress conditions. As a result of pollen developing under this continuous stress cycle, their transcriptomes are predicted to reflect an adaptation to and “memory” of these conditions, as well as responses during the last 1 h HS period. While the mature pollen grains from *Arabidopsis* are desiccated, it is not clear how responsive they are to transcriptome changes during this “dormant state”, and to what degree the HS-changes observed here reflect the final stages of the stress regime, or the history of the stress.

A total of 12 pollen samples were processed with three independent biological replicates for each genotype and growth condition. Transcriptome sequencing was done with a single Illumina flow cell. Expression read counts (Additional file 2) were normalized (Additional file 3) to facilitate a comparison of all 12 samples. More than 21 million reads were obtained for each sample (Additional file 4a), with a principal component analysis (PCA) of the filtered and normalized expression data showing that 87% of the observed variance of the samples can be explained by differences in the stress states. (Principal Component 1, Additional file 4b). The expressed transcript fragments (reads) were aligned to the Araport 11 reference genome [21] for all non-obsolete gene models. While expression of at least three read counts were detected for 24,860 genes (Additional file 2), these results were filtered to identify 14,226 genes showing expression levels deemed high enough for quantification of transcripts in the following 8 gene-type categories annotated by Araport [21]: protein_coding, ara11_novel genes, long_noncoding_RNA, antisense_long_noncoding_RNA, miRNA, other_RNA, small_nuclear_RNA, small_nucleolar_RNA.

Two approaches were used to evaluate purity of pollen samples used for transcriptome comparisons. The first was to use light microscopy to visually inspect pollen samples for non-pollen debris that might have passed through a 70 μm nylon mesh screen during the harvesting protocol. This visual inspection indicated that filtered samples were free of any large fragments of vegetative tissues or non-pollen debris (data not shown).

In the second method (Additional file 5), transcriptomes were evaluated for the relative abundance of selected transcripts, and then compared to previously published pollen transcriptome studies, including both RNA-Seq and microarray data sets [22, 23]. These comparisons were done using two normalization strategies, each providing equivalent results. Data sets for comparison were normalized using either 1) total transcriptome read counts, or 2) a set of four housekeeping genes that appeared to show highly consistent expression levels across all 12 samples in this current transcriptome study (Additional file 5). The relative transcript abundances were evaluated for i) a control group of genes comprising three members of the CNGCs (*CNGC7*, *8*, and *18*), which were previously studied in the Harper lab and known to be essential to pollen fertility and show moderate to low levels of expression [24, 25], and ii) five known photosynthetic marker genes, three corresponding to light harvesting complex genes, and two corresponding to chlorophyll a/b-binding protein genes [26]. Although pollen grains are not considered to be photosynthetic, they contain plastids, with photosynthetic genes showing low basal levels of expression. To use these reference genes to evaluate whether the current study had a level of pollen purity similar to two previous pollen studies [22, 23], we confirmed that the average abundance ratio for three “pollen-specific” *CNGC* reference genes ranged from 1.0 to 2.1 (values of samples here divided by normalized values in [22] or [23]), which suggests that the pollen purity of the current study was equivalent (“1.0”) or even 2-fold more pure (“2.1”). As further support, the transcript abundance for the five different photosynthetic marker genes were all very low, and ranged from being 3-fold less to 1.7-fold higher in the current study compared to [22, 23], respectively. These comparisons indicate that the purity of the pollen in the current study is similar to that of other studies.

To test whether samples from a *cngc16* knockout showed an expected deficiency in full-length *CNGC16* mRNA, individual RNA-Seq reads corresponding to *CNGC16* transcripts were aligned with a reference genome sequence (Additional file 6) using a Web-based tool, Integrative Genome Browser (IGB, bioviz.org). This alignment failed to detect any evidence of full-length transcripts in pollen harboring a *cngc16–2* transfer DNA (*T-DNA*) insertion. Together, the absence of full-length transcripts along with a transgene rescue experiment from Tunc-Ozdemir et al. [20] supports the contention that *cngc16–2* is a true loss-of-function knockout (i.e., null allele).

### WT pollen HSR showed 2102 changes

**Fig. 1:**
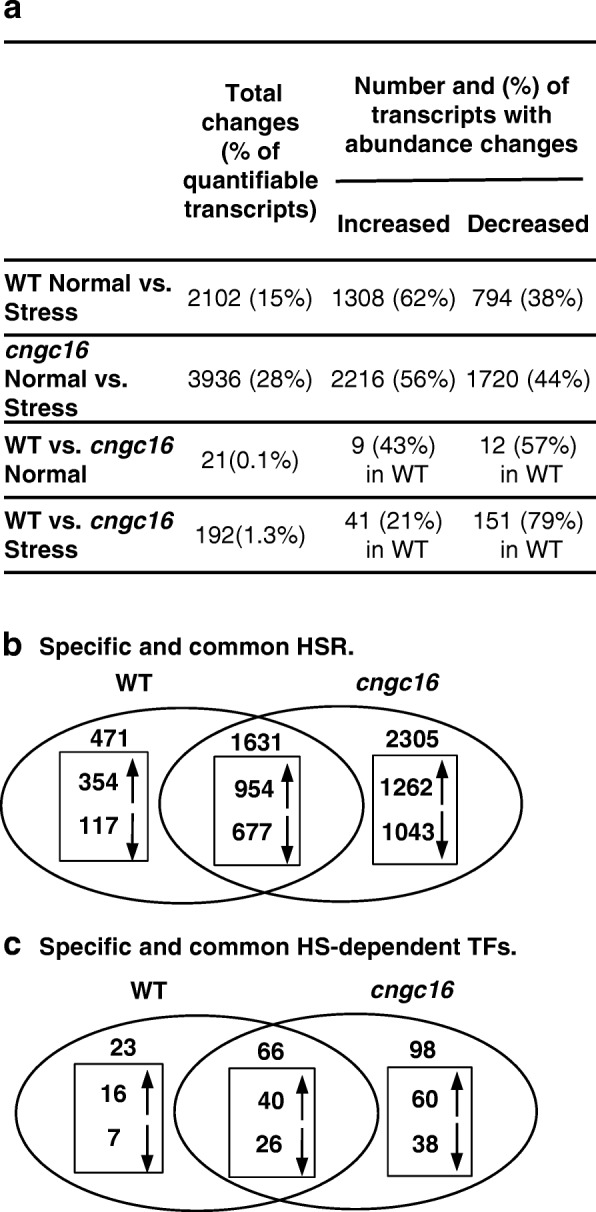
A comparison of WT and *cngc16* transcriptomes reveals numerous differences in their HSRs. **a**. Table shows number of significant changes in transcript abundance out of 14,226 quantifiable transcripts in WT and *cngc16* pollen. **b**. Venn diagram showing number of significant HS-dependent changes that are shared or specific to WT or *cngc16* pollen. **c**. A Venn diagram showing number of significant HS-dependent changes in transcription factors (TFs) that are shared or specific to WT or *cngc16* pollen. All numbers are extracted from Additional file 3. Separate lists of transcript changes between unstressed WT and *cngc16* mutant (21), specific and common HSRs (471, 2305, and 1631), and direct HSR (192) are provided in Additional file 9. Specific lists of TF changes are provided in Additional file 11

To identify genes involved in a WT pollen HSR, we compared normalized transcript abundance profiles for pollen harvested from plants grown with or without a temperature stress regime described in [20]. This comparison showed 2102 statistically significant changes (≥ 2-fold changes with adjusted *p*-value ≤0.01; Fig. 1a; Additional file 3). To confirm the reliability of the RNA-Seq data, four genes were chosen and subjected to a quantitative PCR (Q-PCR) analysis (Additional file 7a, with RNA-Seq results also shown in Table 1). The Q-PCR analysis confirmed relative transcript abundance differences with an overall Spearman Correlation Coefficient computed as 0.72. As expected, correlations were highest for individual examples in which RNA-Seq results showed ≥2-fold differences. When comparing the WT-heat and *cngc16*-heat responses individually, the Spearman Correlation Coefficients were 1.0 and 0.67, respectively. The correlation dropped to 0.02 for the *cngc16* control (normal). We believe this comparison was negatively impacted by the *Myb29* transcripts being undetectable in *cngc16* pollen under control (normal) condition, but still capable of being amplified into a detectable signal using Q-PCR.

**Table 1:** Transcriptome profiles for 33 transcription factor genes with potential relevance to a pollen HSR

Gene ID	Description	WT Norm Avg.	WT Heat Avg.	log2-CHG	Sig. adj *p*-val.	Mut Norm Avg.	Mut Heat Avg.	log2-CHG	Sig. adj *p*-val.	HS- seedling log2- CHG^b^	Ratio of pollen to seedling ^c^
Reference Genes											
AT3G48010	*CNGC16*, cNMP-gated channel	539	733	0.43	N	98^e^	94	−0.08	N	0.23	284
AT5G14870	*CNGC18,* cNMP-gated channel	3741	4158	0.15	N	2989	2620	−0.19	N	−0.08	185
AT5G52640	*HSP90, heat shock protein*	8.3	739	5.40	Y	6.2	489	5.03	Y	5.94	0.010
AT5G59720	*HSP18.2*, heat shock protein	26	309	3.27	Y	29	257	2.89	Y	6.38	0.314
AT3G08770^a^	*LTP6,* Lipid transfer protein	41	108	1.31	Y	153	31	−2.11	Y	−0.53	0.005
AT3G20210^a^	*DELTA-VPE*, endopeptidase	5.7	31	2.08	Y	5.8	3.7	−0.54	N	0.07	2644
AT5G07690^a^	*MYB29,* myb domain protein	1.5	1.0	−0.27	N	< 0.43	13	2.65	Y	−0.89	NA
AT5G66300^a^	*NAC105,* NAC domain	38	1.0	−1.12	N	207	74	−0.88	N	0.15	2
27 transcription factors with heat-altered expression in WT and preferential expression in pollen											
AT3G05860	*AGL45,* MADS-box	1.9	47	3.51	Y	5.0	16	1.28	N	−0.05	14
AT5G37800	*RSL1*, RHD SIX-LIKE 1	15	208	3.45	Y	4.8	101	3.60	Y	NA	43
AT5G58010	*LRL3,* LJRHL1-like 3	40	345	3.00	Y	47	186	1.91	Y	0.48	1308
AT1G49120	*CRF9,* Integrase-type	5.7	50	2.67	Y	6.2	29	1.85	Y	NA	21
AT5G17320	*HDG9,* Homeodomain	6.4	37	2.19	Y	6.8	12	0.56	N	0.24	120
AT3G53790	*TRFL4,* TRF-like 4	3.0	17	1.93	Y	6.9	22	1.39	N	0.19	104
AT4G00120	*IND1,* Basic helix-loop-helix	7.6	33	1.80	Y	6.6	33	1.91	Y	NA	16
AT2G24681	*REM11,* AP2/B3-like	2.6	13	1.71	Y	4.3	12	1.19	N	NA	3
AT5G50480	*NFYC6,* Nuclear factor Y	4.2	16	1.56	Y	3.5	8.8	0.97	N	0.18	270
AT5G60480	*HB26,* Homeobox protein 26	344	876	1.29	Y	331	868	1.33	Y	NA	30246^d^
AT4G26440	*WRKY34,* WRKY DNA-binding	59	147	1.27	Y	61	114	0.86	Y	−0.04	155
AT5G61620	*MYB-like*	51	113	1.10	Y	37	78	1.00	Y	−0.06	185
AT1G26610	C2H2-like zinc finger protein	50	23	−1.06	Y	48	19	−1.25	Y	0.13	8
AT4G16110	*ARR2,* Response regulator 2	3705	1723	−1.07	Y	3475	1282	−1.40	Y	−0.44	8
AT3G22760	*SOL1,* Tesmin/TSO1-like	8947	4051	−1.10	Y	10,247	3080	−1.66	Y	0.04	12
AT5G27090	*AGL54*, AGAMOUS-like 54	179	79.8	−1.12	Y	300	104	−1.47	Y	NA	5382
AT3G57390	*AGL18,* AGAMOUS-like 18	17,513	7358	−1.23	Y	18,821	5458	−1.75	Y	0.06	42
AT2G20110	*TCX6,* Tesmin/TSO1-like	325	120	−1.39	Y	265	52	−2.25	Y	0.20	3
AT5G55020	*MYB120,* myb domain	458	158	−1.42	Y	404	123	−1.59	Y	0.18	61
AT4G08250	*SCL26,* GRAS family	70	23	−1.53	Y	99	25	−1.92	Y	0.10	5
AT4G37940	*AGL21,* AGAMOUS-like 21	124	37	−1.66	Y	186	37	−2.24	Y	0.04	3468
AT5G17430	*BBM,* Integrase-type	62	15	−1.77	Y	57	8.6	−2.31	Y	0.07	77
AT2G37000	*TCP11,* TCP family	128	33	−1.82	Y	99	20	−2.14	Y	0.05	22
AT5G65330	*AGL78,* AGAMOUS-like 78	137	34	−1.90	Y	130	20	−2.51	Y	0.04	301
AT1G72350	*MADS-box*	164	38	−2.01	Y	150	29	−2.26	Y	0.18	402
AT1G66370	*MYB113,* myb domain	13	1.2	−2.41	Y	24	1.5	−3.12	Y	0.17	4
AT5G21120	*EIL2,* Ethylene insensitive	182	23	−2.48	Y	219	20	−2.85	Y	0.48	18
6 transcription factors without a WT HS-response, but still showing a significant > 2-fold difference between *cngc16* and WT											
AT5G24610	cAMP-response element-binding	7.6	16	0.91	N	12	43	1.73	Y	−0.17	0.041
AT2G34440	*AGL29, AGAMOUS-like 29*	6.8	11	0.68	N	< 0.43	< 0.43	0.00	N	0.17	52
AT1G78080	DREB subfamily A-6, *RAP2.4*	26	42	0.61	N	19	106	2.28	Y	1.57	0.002
AT1G10200	GATA-type Zn finger protein	8.32	12.2	0.43	N	10	46	1.85	Y	−0.04	NA
AT1G12260	*NAC 007*, NAC-domain protein	801	1002	0.30	N	529	422	−0.31	N	0.25	9
AT1G10610	bHLH DNA-binding protein	245	190	−0.35	N	521	296	−0.79	N	−0.20	1.4

Eight reference genes are shown at the top, including the four genes marked with (^a^) which were chosen for verification by Q-PCR

^a^Genes used for RNA-Seq validation by Q-PCR

^b^HS-dependent changes in transcript abundance in seedlings (aerial parts) based on publically available data using the AtGenExpress Visualization Tool (AVT) (http://jsp.weigelworld.org/expviz/expviz.jsp) [33] for seedlings exposed to one hour HS at 38 °C. The log2-fold change was calculated based on a comparison of means of normalized values for two heat-stressed and two non-stressed seedling samples

^c^Ratio of expression between pollen and seedling is based on [22]. NA stands for not applicable because it was not possible to calculate based on the information available

^d^Because seedling value was below the limit of detection (< 0.0019), a minimal value of 0.0019 was used in its place as a denominator (0.0019 was the RPKM for ATCG00860 and was the lowest value reported in Loraine et al., 2013 (PMCID: PMC3668042 [22])

^e^cngc16 read counts were restricted to the 5′ end of a truncated transcript (see Additional file 6 and text for more details)

*Norm* Normal, *Avg* Average, log2-CHG log2-fold change, Sig. adj. *p*-val significance based on adjusted *p*-value ≤0.01, *Mut cngc16, N* No, *Y* Yes, *cNMP* cyclic-nucleotide monophosphate, *Ez* Enzyme

Given the ability of RNA-Seq strategies to detect and accurately quantify transcripts with very low abundance, an important question to be addressed on a gene-by-gene basis is whether a transcript with low-abundance has biological importance. It is certainly possible that significant biological impacts during normal development or HS can be associated with very small changes in transcript abundance, or even for transcripts that are below the detection limit of the current study. To give a perspective here on transcripts considered to be “low abundance”, the depth of sequencing in this study allowed for a minimum relative read count after normalization of 0.43 for the WT control (normal) samples. For these samples, the median abundance of all transcripts quantified was 31, or approximately 72-fold higher than the minimum threshold read count. One of the transcripts verified here by Q-PCR was chosen because it corresponded to a relatively low abundance of 1.5 in WT control (normal) conditions and showed a significant HS-dependent increase in abundance in the *cngc16* mutant (i.e., transcription factor gene *MYB29*, Additional file 7a, with RNA-Seq results also shown in Table 1). This example provides confidence that transcripts showing changes near the lower limits of detection in this RNA-Seq data set can still be quantified with statistical confidence. Nevertheless, reliable detection does not address whether a low abundance transcript has an important biological function, or is simply present at near-background levels from “leaky” expression. As evidence here to emphasize the potential biological importance of relatively low abundance transcripts in pollen, we identified two examples of genes, with genetic evidence for a biological function in pollen, and abundance levels near or below our threshold for quantification. For example, at a low expression average of 7.2 in WT control samples, there is a gene encoding a putative acetyl-transferase for which we have genetic evidence for a biological role in pollen HS tolerance, based on a reduced pollen transmission efficiency and gene rescues for two independent *T-DNA* gene disruptions (Harper unpublished). In addition, despite an abundance level that failed to even rise above our threshold for quantification (e.g., 0.43 in WT), there is genetic evidence for a critical pollen autonomous function for AT4G01220 (*MGP4)*, which encodes a sugar nucleotide transferase [27]. Thus, in the context of speculating on gene functions, these two examples provide a precedent that genes with relatively low abundance levels of 7.2 or lower can still have significant biological impacts on pollen fertility.

To evaluate whether the large number of transcriptome changes observed for the WT HSR could be linked to changes with transcription factors, we identified 89 HS-dependent changes associated with transcription factors in WT pollen (i.e.*,* 23 + 66 in Fig. 1c), corresponding to 14% of the 616 transcription factors that were deemed quantifiable in our pollen transcriptomes. Of those, there were 27 showing at least a two-fold higher expression in pollen compared to seedling tissues (Table 1, based on ratios calculated from [22]). These pollen biased transcription factors represent potential regulatory nodes of importance to understanding the pollen-specific features of a HSR.

Heat stress also resulted in an increased abundance of 79 non-protein coding transcripts in WT (Table 2 and Additional file 3) including six microRNAs: *MIR156A, MIR156C, MIR160, MIR836A, MIR831A,* and *MIR780A*. Candidate targets for these microRNAs were determined by psRNATarget (http://plantgrn.noble.org/psRNATarget/, [28]) (see Additional file 8) and included members of the *Squamosa Promoter Binding Protein-Like* (*SPL*) and *Auxin Response Factor* (*ARF*) transcription factor families, which are known to contribute to stress tolerance in vegetative tissues [29] and regulate floral development [30]. Table 2 shows four categories of non-coding RNAs, with examples illustrating the two highest x-fold changes (up and down) for each category. Given that our RNA sample preparations included a selection step for poly-adenylated transcripts, there was an expectation that many of the non-coding RNAs would be selected against and therefore under-represented in our RNA-Seq results. This was corroborated by the observation that from a list of 689 different tRNAs (transcribed by RNA polymerase III), there were only four different transcripts detected with abundance levels that met our stringency thresholds for quantification (Additional file 2). Nevertheless, polyadenylation does occur for some non-coding RNAs that are transcribed by RNA polymerase II [31]. For example, a transcript encoding a miRNA might accumulate as a polyadenylated mRNA before processing into a mature miRNA, and these maturation events could be sensitive to heat stress. Thus, it is possible that some of the non-coding mRNAs identified in Table 2 include transcripts that accumulate before a final processing event. Regardless, their consideration as potential HS-dependent changes are warranted, as the statistical criteria imposed here still provide confidence that changes in their relative transcript abundances were quantified with a reasonable degree of reliability (*p* ≤ 0.01).

**Table 2:** Examples of HS-dependent changes in non-coding RNAs

Gene ID	Description	WT Norm Avg.	WT Heat Avg.	log2- CHG	Sig. adj. *p*-val.	Mut Norm Avg.	Mut Heat Avg.	log2-CHG	Sig. adj. *p*-val.	Ratio of pollen to seedling^a^
Micro RNAs, 6 total for WT, 4 shared by *cngc16*										
AT2G25095	MIR156A	13.30	112.42	2.84	Y	18.26	80.45	1.99	Y	4
AT4G31877	MIR156C	2.64	17.79	2.17	Y	3.54	14.85	1.62	Y	1
AT2G39175	MIR160A	1.89	15.10	2.17	Y	7.75	12.67	0.62	N	3
AT2G25011	MIR836A	4.16	23.65	2.06	Y	7.03	18.07	1.18	N	330
AT2G24103	MIR831A	21.97	8.70	−1.21	Y	48.24	9.15	−2.23	Y	144
AT4G14811	MIR780A	131.10	47.38	−1.41	Y	219.82	66.31	−1.68	Y	52,756
Anti-Sense Long RNAs, 9 total for WT, 7 shared by *cngc16*										
AT1G04533	overlaps AT1G06923	0.37	7.29	2.11	Y	0.00	4.52	1.68	N	NA
AT2G09430	overlaps AT2G43235	303.78	87.30	−1.72	Y	329.94	57.35	−2.41	Y	NA
Long Non-Coding, 40 total for WT, 31 shared by *cngc16*										
AT5G04635	NA	12.13	317.30	4.36	Y	10.99	220.83	3.94	Y	NA
AT5G04815	NA	55.99	8.40	−2.50	Y	57.14	10.29	−2.29	Y	NA
Small Nucleoar and Nuclear RNAs, 10 total for WT, 7 shared by *cngc16*										
AT4G04475	NA	0.00	6.28	2.24	Y	0.00	3.33	1.43	N	NA
AT3G57645	U2.2	15.21	1.26	−2.45	Y	38.85	5.27	−2.44	Y	20
Other RNAs, 14 total for WT, 10 shared by *cngc16*										
AT5G28824	NA	0.00	21.53	3.50	Y	0.00	11.77	2.57	Y	17
AT1G64563	NA	1573.5	300.01	−2.33	Y	1130.83	187.96	−2.52	Y	15

^a^Ratio of expression between pollen and seedling is based on [22]

*Norm* Normal, *Avg* Average, *log2-CHG* log2-fold change, Sig. adj. *p*-val: significance based on adjusted *p*-value ≤0.01, Mut: *cngc16, N* No, *Y* Yes

### The *cngc16* pollen HSR includes 3936 transcriptome changes

To define the transcriptome changes associated with a HSR in *cngc16* pollen, we compared normalized transcript abundance profiles from pollen harvested from mutant plants grown with or without a temperature stress regime in parallel with WT plants discussed above. In *cngc16* pollen, there were 3936 HS-dependent changes (≥ 2-fold changes with adjusted *p*-value ≤0.01; Fig. 1a; Additional file 3), which represents a 1.9-fold increase in the number of changes compared to the 2102 changes that met the same stringency criteria in WT.

### Under control conditions there were only 21 significant differences between WT and *cngc16* transcriptomes

To determine if the *cngc16* knockout mutation resulted in a transcriptome with significant pre-existing differences under control conditions (i.e., “pre-existing condition”), we compared WT and *cngc16* transcript profiles for pollen harvested from plants grown under normal conditions. Only 21 transcripts were significantly different based on the standard threshold criteria used here (≥ 2-fold changes with adjusted *p*-value ≤0.01; Fig. 1a; Additional file 9a). Nevertheless, these 21 examples still included large log2-fold differences that ranged from 3.7 to − 4.2. While the number of pre-existing differences is relatively small, it remains possible that one or more of these 21 differences potentiates a different HSR in the *cngc16* mutant.

### Under HS-conditions, there were 2776 differences between the WT and *cngc16* HS responses

In contrast to the small number of transcriptome differences (i.e., 21) between WT and *cngc16* pollen under normal conditions, there were 2776 differences in a simple contrast comparison between the two lists of genes independently categorized as HS-dependent in WT or *cngc16* (Fig. 1b; Additional file 9b and c). These included 471 transcript changes that were unique to WT (Additional file 9b), and 2305 that were unique to *cngc16* (Additional file 9c). However, to identify the most statistically significant differences, a direct comparison was made between the abundance of transcripts in the WT and *cngc16* HS-transcriptomes. Using the same standard criteria applied above for normal conditions (≥ 2-fold changes with adjusted *p*-value ≤0.01), the HS-transcriptomes showed 192 differences (Additional file 9e, or Additional file 3 column R), of which 10 were already present under normal conditions (i.e., 182 new HS-dependent differences). This represents an approximate 9-fold increase in the differences between WT and *cngc16* transcriptomes in response to a HS. Using a less stringent cut-off criteria that allowed for any difference between WT and *cngc16* with a *p*-value greater than 0.05, there was a much greater number of 1531 differences between the two HS-transcriptomes, or an approximate 13-fold increase compared to control (normal) conditions. Thus, while there are potentially as many differences as there are similarities between the WT and *cngc16* HSRs, a smaller subgroup of 182 HS-dependent differences can be categorized with a greater degree of statistically confidence (see Additional file 3, column R or Additional file 9e).

To highlight some of the most dramatic differences, Table 3 shows the top 23 examples of HS-dependent changes in WT that displayed the largest magnitude differences between WT and *cngc16* under HS. These differences were analyzed for potential functional associations using STRING [32], revealing a cluster of seven genes that were classified as under-responsive to HS in *cngc16* (Table 3; Additional file 10). While the functional significance of this under-responsive cluster is not known, it includes 4 genes related to cell walls and membrane dynamics. Interestingly, cell walls and membrane dynamics are the most frequent functional category for all of the genes in Table 3 (half of the top 20 largest differences).

**Table 3:** HS-dependent changes in WT that were most different in *cngc16* under HS

Gene ID	Description	Net-work ^a^	Function based on S (String), MM (MapMan), T (TAIR)	WT Heat Avg.	*cngc16* Heat Avg.	log2- CHG	Sig. adj. *p*-val.	Seed-ling HS log2- CHGb	Ratio pollen to seed-ling ^c^
Over-responsive in *cngc16* compared to WT under HS									
AT4G33790	FAR3, CER4, acyl CoA reductase	B	Cell Wall, Cuticular Wax ^S^	5.6	30.4	2.1	0.001	0.16	0.03
AT3G05650	Receptor-like protein 32, RLP32		Biotic Stress Signaling ^S^	20.4	73.2	1.7	0.009	−0.58	< 0.01
AT3G44310	Nitrilase 1		Secondary Metabolism ^MM^	54.1	146	1.4	< 0.001	−0.43	< 0.01
AT5G59320	Lipid transfer protein 3	C	Membrane Dynamics ^S^	131	366	1.4	0.004	0.32	0.06
AT5G25280	Serine-rich protein		Unknown ^S,MM, T^	146	376	1.3	0.010	2.05	0.01
AT5G59613	ATP synthase		ATP Synthase ^T^	31.5	78.3	1.2	0.001	NA	0.08
AT5G15960	Stress-responsive protein (KIN1)		Stress Protein, Antifreeze ^T^	58.4	139	1.2	0.005	0.09	0.00
AT5G59310	Lipid transfer protein 4	C	Membrane Dynamics ^S^	377	879	1.2	0.004	0.32	0.17
AT2G24940	MAPR2 steroid-binding protein 3		Membrane Dynamics ^S^	40.9	91.8	1.1	0.009	0.15	0.02
AT3G04120	GAP dehydrogenase C subunit 1	B	Glycolysis ^MM^	160	338	1.0	0.001	0.12	0.01
AT1G07590	Tetratricopeptide repeat (TPR)-like		Gene Expression ^MM^	66.5	142	1.0	0.006	0.15	0.02
Under-responsive in *cngc16* compared to WT under HS									
AT2G20110	Tesmin/TSO1-like with CXC domain		Gene Expression ^MM^	120	51.6	−1.2	0.002	0.20	2.60
AT1G49490	LRX9, Leucine-Rich Repeat/Extensin 9		Cell Wall Functions ^MM^	149137	61672	−1.2	< 0.001	0.09	702
AT2G46192	Non-coding RNA, “other_rna”		Gene Expression ^MM^	91.0	33.6	−1.4	< 0.001	NA	2.57
AT5G17320	HDG9, homeodomain GLABROUS 9		Gene Expression ^MM^	37.0	11.6	−1.5	0.010	0.24	120
AT4G12870	Lysosomal thiol (GILT) reductase	A	Unknown ^S,MM, T^	20.3	5.8	−1.6	0.007	NA	0.02
AT3G08770	Lipid transfer protein 6	A	Membrane Dynamics ^S^	108	31.2	−1.6	< 0.001	−0.53	< 0.01
AT1G56100	Invertase/pectin methylesterase inhibitor	A	Cell Wall Functions ^MM^	21.7	3.2	−2.3	< 0.001	0.02	< 0.01
AT2G46960	CYP709B1, cytochrome P450	A	Stress Responsive P450 ^S^	17.8	2.3	−2.3	0.002	0.29	6.63
AT5G47350	Alpha/beta-Hydrolases superfamily	A	Membrane Dynamics ^T^	43.8	6.0	−2.5	< 0.001	0.07	< 0.01
AT3G20210	Delta vacuolar processing enzyme	A	Protein Degradation ^S^	31.0	3.7	−2.6	< 0.001	0.07	2644
AT4G15750	Invertase/pectin methylesterase inhibitor	A	Cell Wall Functions ^MM^	51.7	5.2	−2.9	< 0.001	0.22	17.1
AT1G26240	Proline-rich extensin-like family		Cell Wall Functions ^MM^	14.4	0.4	−3.2	< 0.001	0.09	0.50

^a^Network group analysis was done using STRING [32]

^b^HS-dependent changes in transcript abundance in seedlings (aerial parts) based on publically available data using the AtGenExpress Visualization Tool (AVT) (http://jsp.weigelworld.org/expviz/expviz.jsp) [33] for seedlings exposed to one hour HS at 38 °C. The log2-fold change was calculated based on a comparison of means of normalized values for two heat-stressed and two non-stressed seedling samples

^c^Ratio of expression between pollen and seedling is based on [22]. NA stands for not applicable because it was not possible to calculate based on the information available

Functional annotation based on S (STRING, [32]), MM (MapMan, [71]), and T (TAIR, [64]), as noted

### The WT and *cngc16* HSRs showed 121 differences corresponding to transcription factors

A focused comparison of HS-dependent transcript changes for transcription factors was done to assess whether differential expression of transcription factors might contribute to the greater number of overall transcript changes in *cngc16*. While 66 HS-dependent transcription factor changes were common to *cngc16* and WT pollen, there were 121 (1.8-fold more) changes categorized as potential differences (Fig. 1c; Additional file 11). However, in a direct comparison between the HS-transcriptomes of WT and *cngc16*, there were only seven transcription factor differences that satisfied a more stringent statistically criteria of ≥2-fold difference with an adjusted *p*-value ≤0.01 (see Table 1). Only one of these seven showed a significant HS-dependent change in both WT and *cngc16* pollen (AT2G20110). This transcription factor also showed preferential expression in pollen compared to seedlings (listed in middle section of Table 1). The other six transcripts either just showed HS-dependent changes in *cngc16* pollen, or were simply significant differences that were independent of a stress condition (bottom of Table 1). Regardless, any small changes in the abundance of transcription factors between WT and *cngc16* could easily cause the larger number of downstream transcriptome differences observed under a HSR.

### HS-dependent transcriptome changes for Ca^2+^-signaling related genes

**Fig. 2:**
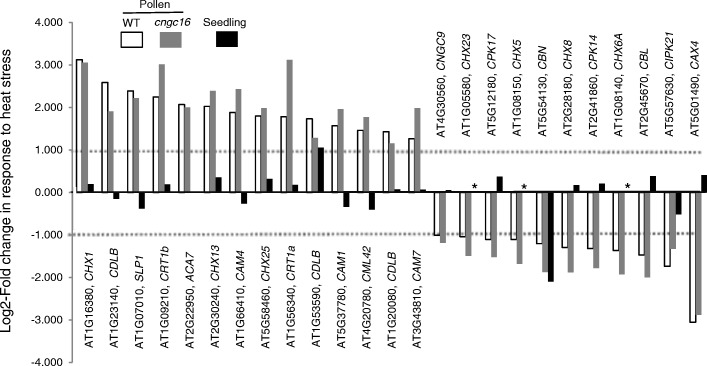
HS-dependent changes for 25 Ca^2+^-signaling related genes. Graph shows selected examples of HS-dependent log2-fold abundance changes for transcript encoded by 25 genes related to Ca^2+^-signaling. The 25 examples were selected as the most statistically significant changes in WT pollen (open bar) based on adjusted *p-*values. Corresponding transcript abundances from *cngc16* mutant (grey bar) and aerial parts of seedlings (black bar) are shown for comparison. HS-dependent changes in transcript abundance in seedlings (aerial parts) was based on [33]. Complete list of Ca^2+^-related genes with putative functions (total 230) is provided in Additional file 12. CHXs are cation/H^+^-exchangers. CBLDs are Ca^2+^-dependent lipid-binding (CaLB domain) family protein. SLP is Calcineurin-like metallo-phosphoesterase superfamily protein. CRTs are calreticulins. ACA is autoinhibited Ca^2+^-ATPase pump. CAMs are calmodulins. CML is calmodulin like. CNGC is cyclic nucleotide gated channel. CPKs are Ca^2+^-dependent protein kinases. CBN is Ca^2+^-binding endonuclease/exonuclease/phosphatase family. CBL is calcineurin B subunit-like protein. CIPK is calcineurin B-like (CBL)-interacting protein kinase. CAX is cation exchanger. * indicates absence of probeset in microarray experiment

Because *CNGC16* is thought to function in both creating and sensing Ca^2+^-signals, a transcriptome comparison was done to evaluate whether WT and *cngc16* pollen showed significant HS-dependent changes for a subset of genes associated with Ca^2+^-signaling (Ca^2+^-signaling related genes). Pollen expression was detected for 230 Ca^2+^-signaling related genes (Additional file 12). Of those, 40 (or 17%) showed significant changes in a WT HSR (Fig. 2). In a direct comparison between the WT and *cngc16* HS-transcriptomes, there were no examples (except *CNGC16*) of any differences that met our standard criteria of ≥2-fold change with an adjusted *p*-value ≤0.01. This suggests that in the context of feedback networks that regulate Ca^2+^-signaling related genes, the loss of CNGC16 did not have a significant impact.

To evaluate whether Ca^2+^-signaling related genes showed similar or different HSRs between pollen and vegetative tissues, a comparison was made with publicly available data sets obtained from AtGenExpress (http://jsp.weigelworld.org/expviz/expviz.jsp; [33]). While caution is required in making comparisons between experiments done with different HS conditions and transcript profiling technologies, the HS-dependent changes in pollen and seedlings appeared very different. For the subset of 40 Ca^2+^-signaling related genes that showed a significant change in WT pollen, only two genes showed an analogous HSR in seedling tissues (*Calcium-dependent lipid-binding (CaLB domain) family protein*, AT1G53590 and *Calcium-binding endonuclease/exonuclease/phosphatase family*, AT5G54130) (Fig. 2). Of the remaining 38, half did not even show a transcript abundance change in the same up or down direction. Thus, there was only a 5% overlap between HS-dependent changes in Ca^2+^-related genes detected in pollen and seedlings.

## Discussion

While thermotolerance in plants has been widely studied, relatively little is known about specific effects of temperature stress on pollen [7, 11, 12]. Nevertheless, fertilization during plant reproduction is highly sensitive to hot and cold temperatures, and pollen is often considered a weak-link in reproductive stress tolerance [1–6].

This study provides a reference data set that identifies at least 2102 transcriptome changes associated with a HSR in WT *Arabidopsis* pollen (≥ 2-fold changes with adjusted *p*-value ≤0.01; Fig. 1a; Additional file 3). In addition, a parallel comparison with pollen from a *cngc16* knockout mutant provides evidence that the HS-sensitivity caused by a *cngc16* mutation [20] could be due in part to a defect in reprogramming the pollen transcriptome during a HSR.

### HSRs in pollen and vegetative tissues have important similarities and differences

A potentially important conceptual difference between stress-tolerance programs in pollen and vegetative cells is that vegetative cells can often respond to a HS by down-regulating metabolism into a “survival mode”, and thereby wait for better growth conditions, whereas pollen tubes are often under temporal constraints in which there is a limited window of time to find and fertilize ovules. Because pollen must grow as fast as possible to successfully compete and “win a race” to fertilize a limited number of ovules, for some pollen types, a stress-response is postulated to include important differences compared to other cell-types in which the simplest solution to a HS is to down-regulate metabolism and wait for more optimal growth conditions.

The 2102 HS-dependent transcriptome changes observed here for WT pollen represent a dramatic reprogramming of at least 15% of the transcriptome (at ≥2-fold with adjusted *p*-value ≤0.01). The full extent of this reprogramming is most likely higher, with an estimate of 24% using a lower threshold criteria that includes smaller magnitude changes (i.e., less than two-fold), and/or abundance changes with a slightly more permissive *p*-value (e.g., ≤ 0.05 instead of ≤0.01). This extensive reprogramming has been observed in other studies in both plant and non-plant systems [34–38].

Many of the expected HS-dependent changes were observed here in pollen, such as an increased abundance of transcripts corresponding to heat shock transcription factors (HSFs*,* e.g. *HsfA2*), small heat shock proteins (HSPs), *BCL-2-associated athanogene 6* (*BAG6*)*,* WRKY transcription factors, *Multiprotein Bridging Factor 1c* (*Mbf1C*), dehydrogenases, phospholipases, and hormone-responsive transcription factors involved in ethylene (AT5G47230) and auxin (AT3G23050) signaling [20, 39–43]. Among the common HSRs were also antioxidant enzymes such as ascorbate peroxidase 2, peroxidase, and catalase [44–46].

Despite similarities with other HSRs, the following four observations create an expectation of important differences between a HSR in pollen compared to other plant cells. First, of the 89 transcription factors that showed a HS-dependent change in WT pollen, 84 (or 94%) failed to either be detected or show a similar HS-response in aerial parts of *Arabidopsis* seedlings (Additional file 11), based on a comparison with publicly available microarray data in AtGenExpress (http://jsp.weigelworld.org/expviz/expviz.jsp; [33]).

Second, pollen failed to show a HS-dependent change for 96% of a group of 67 genes that were curated as multi-stress regulated genes based on a comparison of nine abiotic stresses, including HS in *Arabidopsis* root and shoots [47]. Of these 67 genes, transcripts from only 19 (less than half) were detected in pollen (Additional file 13). Of those 19, only three showed a significant HS-dependent change in pollen, and of those three, two actually showed an opposite change in abundance compared to a vegetative HSR. The only example that showed a parallel HS-dependent decrease was AT1G05400, which is annotated as encoding a hypothetical protein.

A third observation was the failure to see expected changes in abundance of transcripts encoding SPL transcription factors (*Squamosa Promoter Binding Protein-Like*). In vegetative tissues, there is evidence that a HS-dependent increase in *MIR156* triggers a decrease in transcripts for multiple SPL transcription factors [29, 48]. While the pollen HSR here also showed a HS-dependent increase in transcripts harboring *MIR156a* and *c* (seven to four-fold, respectively), a corresponding decrease in target abundance was not observed for any of the expected *SPL* genes (Additional file 8). In a second similar example*,* pollen showed a HS-dependent increase in transcripts harboring *MIRNA160,* but without a significant decrease detected in predicted regulatory targets (Additional file 8). These examples suggest that *MIR156* and *MIR160A* might function differently during a HSR in pollen compared to other tissues.

A fourth observation was the poor correspondence between pollen and seedlings for HS-dependent responses among a subset of 230 pollen-expressed genes that are implicated in sensing or creating Ca^2+^-signals (Additional file 12). Of 40 Ca^2+^-signaling related genes with transcripts showing a HS-dependent change in WT pollen, there were only two examples of a similar response in vegetative tissues (Fig. 2). Likewise, in a subset of 23 HS-dependent changes in WT that were most different in *cngc16* (Table 3), there was only one that was a potential HS-gene in seedlings, AT5G25280, a gene with unknown functions. Thus, together these four transcriptome-based observations above provide strong evidence that a HSR in pollen has significant differences compared to other cell types in plants.

### A HSR in *cngc16* pollen was significantly different than WT

Results here provide evidence that the HSR in *cngc16* pollen is significantly different than WT. First, in a simple tally of genes categorized as HS-dependent in WT and *cngc16*, there were 2776 differences, with *cngc16* pollen showing 1.9-fold more differences compared to WT (Fig. 1a; Additional file 3). Based on a more rigorous statistical analysis, a subset of 192 genes were found to have a ≥ 2-fold difference (with adjusted *p*-value ≤0.01) in a direct comparison of the HS-transcriptomes from WT and *cngc16* (Additional file 3, column R and Additional file 9e).

While it is not yet clear which transcriptome differences between WT and *cngc16* might have biological importance, some examples from the subgroup of the 192 most statistically significant differences include the following. First, there were seven transcription factors, of which one failed to be detected in the *cngc16* transcriptome under HS or control (normal) conditions (AT2G34440, *AGAMOUS-like 29*). Because transcription factors regulate the expression of other genes, any transcription factor differences between WT and *cngc16* could potentially amplify differences throughout the transcriptome during a HSR.

Second, in evaluating the 23 HS-dependent changes in WT that were most different in *cngc16* (Table 3 and Additional file 10), the two most frequent functional categories were cell wall and membrane dynamics. In the context of cell wall, *FAR3* (*Fatty Acid Reductase 3*, AT4G33790) showed increases in transcript abundance in *cngc16* pollen that were opposite to WT. While FAR3 is involved in cuticular wax production in leaves [49], and is essential for pollen fertility [50], its potential importance to a HSR in pollen has not been investigated.

Another noteworthy example in the cell wall category was a gene encoding Leucine-Rich Repeat/Extensin 9 (*LRX9*, AT1G49490,). Studies on loss-of-function knockouts for members of this extensin subfamily have recently provided evidence for a critical role of these proteins in pollen tube growth [51–54]. Thus, a HS disruption in the expression of these, and other cell wall related genes, could potentially affect the structural dynamics of the cell wall and the ability of pollen tubes to grow and fertilize ovules.

In the context of membrane dynamics, there were three examples of genes encoding lipid transfer proteins (LTPs). While the specific biochemical activities of these proteins and their importance to a HSR is not understood, there is evidence that overexpression of an LTP in potato can protect cell membrane integrity under multiple stress conditions, including heat [55]. In addition, there are many studies showing the importance of regulating lipid biogenesis pathways for cells to adapt to temperature stresses [56]. As a specific example for pollen, a double knockout of lipid fippases *ala6* and *ala7* in *Arabidopsis* results in a stress-dependent pollen sterility [57] under the same temperature stress regime used here.

### Role of CNGC16 in HSR

There are two general models to explain the function of CNGC16 in mediating a normal HSR. The first is a direct role of CNGC16 as part of an ion signaling pathway that functions in sensing or responding to HS. For example, a HS-triggered increase in cyclic-nucleotide monophosphate (cNMP) concentrations could activate CNGC16 and generate a cytosolic Ca^2+^ signal. While CNGC16’s ion selectivity properties have not been corroborated, a Ca^2+^ conductance has been suggested based on analogy to other closely related homologs [20]. For example, there is electrophysiology evidence for Ca^2+^ conductance from a study on a closely related *cngc6* knockout [58]. In this case, CNGC6-mediated Ca^2+^-transients are thought to be critical to establishing vegetative HS-tolerance. In addition, CNGCa and CNGCd in a moss *Physcomitrela* have been implicated in modulating Ca^2+^ signals during a HSR [59]. Furthermore, a trio of isoforms closely related to CNGC16 in *Medicago truncatula* (CNGC15a, b, and c) have been implicated in controlling an elicitor-induced Ca^2+^ oscillation associated with the nucleus [60]. These *Medicago* homologs appear to be localized to the nuclear envelope, raising the potential that a HS-dependent Ca^2+^ signal in pollen might also be associated with the ER or nuclear envelope instead of the plasma membrane. However, regardless of subcellular location, a potential CNGC16-mediated Ca^2+^ signal could alter numerous cellular enzymes and structures, including the activity of key transcription factors important to a HSR.

A second alternative model is that a loss of CNGC16 might result in a cell with a pre-existing condition in which mutant cells are “unhealthy” and therefore less-able to sense or respond to multiple stresses, including a HS. While the transcriptome analysis here identified at least 21 differences that might contribute to an unhealthy pre-existing condition, it is not yet clear if any of these differences are actually responsible for the HS hypersensitivity of *cngc16* pollen.

**Fig. 3:**
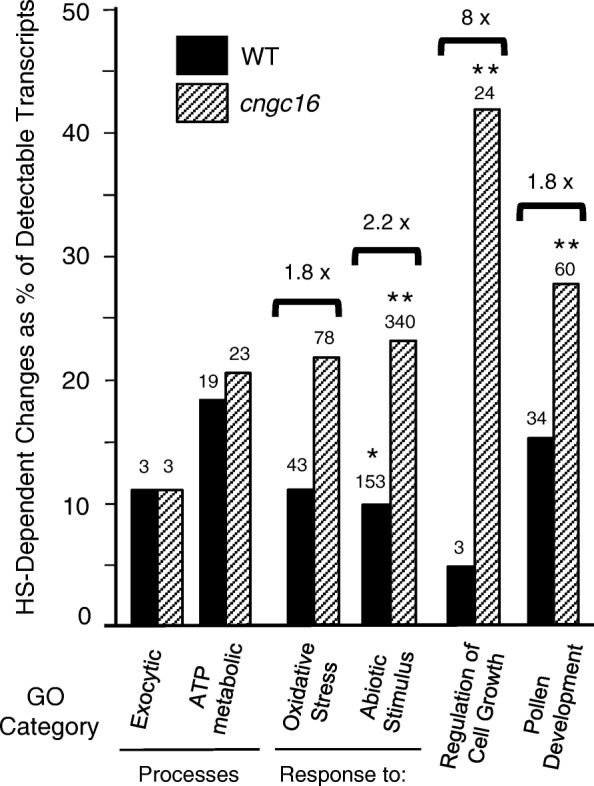
A GO (Gene Ontology) classification reveals similarities and differences between HS-triggered transcriptome changes in WT and *cngc16* pollen. The GO analysis for Biological Process using PANTHER [61] was conducted for 2102 and 3936 HS-dependent changes in WT and *cngc16* mutant (Additional file 14). The number of annotated genes from the input list that were mapped into a particular category is shown above each bar. Brackets show X-fold difference between *cngc16* and WT. The categories shown are exocytic process (GO:0140029), ATP metabolic process (GO:0046034), response to oxidative stress (GO:0006979), response to abiotic stimulus (GO:0009628), and pollen development (GO:0009555). * and ** indicate enrichment in the GO category with *p*-value ≤0.05 and ≤ 0.01, respectively. A more comprehensive comparison of 5053 different GO categories is provided in Additional file 14

Regardless of a direct or indirect mechanism to explain why a *cngc16* mutation results in pollen with hypersensitivity to HS, the HS-transcriptome response in *cngc16* was clearly different than WT (e.g., Table 3, and Additional file 3, column R). In addition to the large number of differences, the nature of those differences suggests several possible mechanistic explanations for hypersensitivity, as revealed by comparing transcript changes in WT and *cngc16* in the context of Gene Ontology (GO-term) classifications (e.g., Biological Process using PANTHER [61]) (Fig. 3 and Additional file 14). First, the *cngc16* mutant showed a 1.8 to 2.2-fold increase in the number of HS-dependent changes categorized as responses to oxidative stress or abiotic stimulus. This increase is consistent with a model in which *cngc16* pollen might be less-capable than WT in mitigating a ROS increase, which often occurs under stress situations. Second, *cngc16* pollen showed an 8-fold greater number of differences in “regulation of cell growth”, and a 1.8-fold greater number of genes related to pollen development. These examples are consistent with a model in which *cngc16* pollen are less-able than WT during a HSR to stabilize transcript levels associated with critical developmental programs related to cell growth in general, and pollen development in particular. While the *cngc16* HSR compared to WT showed differences in more than 80% of 2380 different GO categories (Additional file 14), Fig. 3 includes two examples of controls in which transcript profiles for WT and *cngc16* were similar (e.g., ATP metabolic processes and exocytic processes). A separate GO analysis (Additional file 15) was also done for the subset of 192 genes identified here showing the greatest differences in a direct comparison of *cngc16* and WT under HS (Additional file 9e, or Additional file 3 column R). Under Biological Process, more than two thirds of the over-represented genes belonged to just two categories, metabolic process (GO:0008152) and cellular process (GO:0009987). In a subset of the 20 HS-dependent changes in WT that were most different in *cngc16* (Table 3 and Additional file 10), the two most common categories were cell wall and membrane dynamics.

Thus, HS appears to differentially perturb a large number of the transcriptome homeostasis networks in *cngc16* pollen, with potential functional implications for a wide range of cellular and metabolic processes, including response to oxidative stress, regulation of cell growth, membrane dynamics and cell wall integrity. In this context, the simplest model to explain the HS-hypersensitivity of *cngc16* pollen is that mutant cells are simply more vulnerable to losing control over multiple cellular systems, of which a failure with one or some combination make it impossible to carry out a very complicated development program.

## Conclusions

The HS-transcriptome comparisons here provide new insights into a temperature-stress response in WT pollen. While pollen exhibit many of the same HS-dependent changes characteristic of vegetative tissues, evidence here suggests many important differences. For example, of the 89 transcription factors that showed a HS-dependent transcript abundance change in WT pollen, 94% failed to show a similar HSR in aerial parts of *Arabidopsis* seedlings (Additional file 11). Importantly, 27 of these transcription factor genes are expressed primarily in pollen (Table 1), and therefore represent regulatory nodes of potential importance to understanding pollen-specific features of a HSR.

A comparison of pollen from WT and a heat-sensitive *cngc16* mutant showed only a small number of differences under control (normal) conditions (21), with differences increasing by at least 9-fold under HS. Given that more than 15% of the pollen transcriptome showed changes in response to a HS, it will be difficult to determine which specific differences in *cngc16* pollen represent a potential cause of hypersensitivity, or simply arose as symptoms of a mutant cell’s inability to cope with HS. Nevertheless, results here suggest that *cngc16* pollen have a defect in enacting or maintaining an appropriate HS-transcriptome response. Together, these results support a model in which reprogramming the pollen transcriptome for HS-tolerance is dependent on the proper functioning of a specific *Cyclic Nucleotide-Gated cation Channel, CNGC16.*

## Methods

### Plant material and growth conditions

The *cngc16–2* (*SAIL_726_B04*, seed stock no. 91) mutant carries a glufosinate (Basta) resistance marker in the *T-DNA* insertion [20]. Following sterilization, seeds from both *Arabidopsis* Columbia WT (Col-0) and the *cngc16–2* mutant were sown on 0.5× Murashige and Skoog (MS) medium (pH = 5.7) containing 1% agar with or without Basta (10 μg ml^− 1^). After 48 h of stratification at 4 °C, seedlings were grown at room temperature under constant light for 24 h for 10 days. The resulting seedlings were then transferred to soil (Sunshine SMB-238; Hummert). Unless otherwise stated, seedlings were grown until maturity under non-stress (control/normal) conditions in a growth chamber (Percival Scientific, Inc., http://www.percival-scientific.com/) with a photoperiod of 16 h of light and 8 h of dark at 22 °C, 40% humidity, and light intensity of 125 μmol m^− 2^ s^− 1^.

### Stress condition

The stress regime with hot days and cold nights was performed as described in [20] and diagramed here in Additional file 1. Briefly, flowering plants grown under non-stress (control/normal) condition were transferred to a hot and cold stress-regime chamber and grown for one week before harvesting pollen. Pollen were harvested at the end of a 1 h HS-period at 40 °C (noon) following a gradual temperature rise from − 1 °C starting at 6 AM.

### Pollen collection

For both the WT and mutant, three biological replicates per condition were used. Both HS and control (normal) pollen samples were harvested at midday. To purify pollen, open flowers were cut, vortexed for 10 to 20 s in a 50 ml centrifuge tube containing water, and filtered through a 70 μm nylon mesh (Becton Dickinson and Company). The pollen passing through the filter was centrifuged into a pellet for 30 s at 14000 rpm. The supernatant was discarded, and pollen pellets were frozen in liquid nitrogen and stored in − 80 °C.

### RNA isolation, library preparation, and sequencing

Total RNA was extracted from pollen samples using Plant Mini Kit (Qiagen, Invitrogen), including an optional cleaning step using RNase-free DNase to eliminate genomic DNA contamination. One microgram of total RNA from each sample was sent on dry ice to the UCLA Neuroscience Genomic Core (UNGC) (Los Angeles, CA, USA) for library preparation using the TruSeq RNA kit (Illumina, San Diego, U.S.A) and sequencing. The RNA samples were quantified using the RiboGreen assay (ThermoFisher Scientific), and the quality of the RNA was checked by the Agilent TapeStation 2200. For each library, one microgram of total RNA was poly-A selected using oligo-dT magnetic beads, fragmented, and cDNA synthesized with random primers. Double-stranded cDNA was phosphorylated, and A-tailed followed by adapter ligation, PCR amplification, and sequencing. The cDNA libraries were multiplexed and run on a single lane. Paired-end (PE) sequencing was performed on an Illumina HighSeq2500 with two separate runs, the first generating sequences of 2 × 50 bp length and the second generating 2 × 69 bp read pairs.

Both ends of paired sequences were trimmed using Trimmomatic, version 0.36 [62] to remove sequences containing Illumina sequencing adapters, low-confidence bases (phred Q < 5), and sequences with length < 35 nucleotides. Sequence quality was measured and visualized before and after trimming using FastQC, version 0.11.5 (http://www.bioinformatics.babraham.ac.uk/projects/fastqc/). To verify the reverse/forward strand orientation expected of read pairs produced by TruSeq Stranded mRNA library preparation, subsets of 10,000 read pairs were randomly selected from trimmed read files with seqtk, version 1.0-r82 (https://github.com/lh3/seqtk), and compared to Araport 11 cDNA sequences [21] with BLASTN search via the command-line ncbi-blast+ (v2.5.0) application [63]. The plus/minus strand orientations of the tabular results were used to verify library forward/reverse orientations to correctly configure alignment tools.

The filtered sequence pairs were aligned to the Araport 11 version [21] of the TAIR10 *Arabidopsis thaliana* reference genome sequence [64], which was indexed in combination with coordinate information for positions of all exons and splice sites of transcribed genes annotated in the Araport 11 reference set, version 1.10.4 (released 06/2016) [21], using the spliced-read alignment tool HISAT2, version 2.0.5 [65]. Alignments produced by HISAT2 were converted to the binary BAM format and sorted with samtools, version 1.3.1 [66]. From these alignments, the number of read pairs aligned to known *Arabidopsis* genes was calculated using the FeatureCounts tool within the subread package, version 1.5.1 [67]. Alignments were counted once per pair, summarized at gene loci features, and read pairs with reported alignments to multiple loci were excluded from count totals. With at least three read counts set as a minimum threshold-limit for detection, there were 24,860 transcripts identified with correspondence to gene models or genomic features, excluding any matches to obsolete loci (Additional file 2).

To identify a subset of detectable transcripts deemed appropriate for quantification, two filtering steps were used. First, transcripts were excluded if they were categorized as rRNAs, tRNAs, transposons, or pseudogenes. The remaining categories annotated in Araport were retained for further processing: protein coding, ara11_novel genes, long_noncoding_RNA, antisense_long_noncoding_RNA, miRNA, other_RNA, small_nuclear_RNA, and small_nucleolar_RNA. To eliminate transcripts with expression levels considered too low for reliable quantification, transcripts with less than 10 fragments (counts) observed in at least two biological replicates in any condition were excluded. This left 14,226 transcripts for downstream analyses (Additional file 3). Data were normalized using the standard median ratio method for RNA-Seq data [68]. Principal component analysis (PCA) was performed on the normalized and filtered zero-centered counts per million data using singular value decomposition to validate clear separation between the different conditions (Additional file 4).

### Differential gene expression

Differential gene expression between the four conditions was examined using DESeq2 [68]. Four comparisons including WT_heat vs. WT_control (normal), *cngc16*_heat vs. *cngc16*_control (normal), *cngc16*_control (normal) vs WT_control (normal), and *cngc16*_heat vs WT_heat were considered using simple contrasts. A multiple testing correction was performed within each of the four comparisons to adjust for the false discovery rate [69]. Genes with ≥2-fold changes and adjusted *p*-value ≤0.01 were considered to meet the standard significance threshold for this study.

### Validation of RNA-Seq data by quantitative PCR (Q-PCR)

To validate RNA-Seq data, the transcript levels of four genes (Additional file 7) were examined by quantitative PCR (Q-PCR). Three of these four genes were chosen because they showed significant HS-dependent changes for the WT and or *cngc16* samples in the RNA-seq analysis. The fourth gene was expressed at very low levels with a level of variation that made it a non-significant change. The same RNA samples used for RNA-Seq were used for verification of selected transcript changes using Q-PCR. First strand cDNA was synthesized using one microgram of total RNA via iScript cDNA Synthesis Kit (Catalogue#170–8891; Bio-Rad laboratory). A fraction (0.14 μg) of the cDNA was used as template in a 20 μL Q-PCR reaction mixture using SsoFast Evagreen Supermix (Catalog#172–5201; Bio-Rad laboratory) following the manufacturer’s instructions. Primer sequences used are shown in Additional file 7. Two reference genes *cyclin p2;1* (*CYCP2;1*: AT3G21870) and *UBIQUITIN 7* (*UBQ7*: AT2G35635) were chosen based on their minimal variation in the RNA-Seq analysis among all 12 experimental samples tested. The four other genes were selected to test examples of different patterns of changes observed in a comparison of WT and *cngc16* transcriptomes. These included two transcription factors *NAC105* (AT5G66300) and *MYB29* (AT5G07690), as well as *lipid transfer protein 6* (*LTP6*; AT3G08770) and *delta vacuolar processing enzyme* (*DELTA-VPE;* AT3G20210). Transcript abundance was quantified by Q-PCR (CFX96; Bio-Rad laboratory) with a separate normalization to the two different reference genes. The Q-PCR conditions were as follows: 30 s at 95 °C for enzyme activation, 39 cycles of 95 °C for 10 s for denaturing, and 25 s at 60 °C for annealing/extension.

A fold-change was calculated for each gene (normalized separately to each of the two reference genes *UBQ7* and *CYCP2*) in relation to the expression of the WT control (normal) using the 2^-ΔΔCT^ method [70] (Additional file 7). Based on all conditions and comparisons, the Spearman Correlation Coefficient between the Q-PCR and RNA-Seq expression values was computed as 0.72.

### Gene ontology (GO)

Differentially expressed transcripts showing ≥2-fold changes and adjusted *p*-values ≤0.01 were analyzed using PANTHER [61]. Specifically, a statistical overrepresentation test (release 2017–04-13) was performed with the GO Biological Process Complete Annotation Data Set and a Bonferroni correction for multiple testing. The PANTHER Version 12.0 (release 2017–07-10) and Gene Ontology (GO) database (release 2017–08-14) were used. The test was based on the *Arabidopsis* genome of 27,060 annotated genes (released 07/2016).

### Association network analysis

The top 23 examples of HS-dependent changes in WT with the largest magnitude differences between WT and *cngc16* under HS were analyzed for potential associations or interactions using STRING version 10.5 [32] and MapMan version 3.6.0.[71].

## Additional files

Additional file 1: Hot/Cold stress cycle. Diagram showing the Hot/Cold stress-cycle used here for growing plants from which pollen samples for RNA-Seq experiment were harvested at the end of HS-peak at 40 °C. See Methods and [20] for more details. (PPTX 50 kb)

Additional file 2: Raw expression counts for WT and *cngc16* pollen with and without HS. Read counts generated via FeatureCounts, see Methods) for all 12 replicates are shown before normalization and exclusions. (XLSX 4358 kb)

Additional file 3: Normalized transcript expression counts for WT and *cngc16* pollen with and without HS. Expression counts from Additional file 2 were subjected to exclusions/filters and normalized as described in methods. Expression data from other transcriptome studies (microarray and RNA-Seq) were added to the table for comparisons. These included an RNA-Seq data set for WT pollen and seedlings from Loraine et al. 2013 (PMCID: PMC3668042 [22]), two pollen microarray experiments from Qin et al. 2009 (PMCID: PMC2726614 [23]) and Borges et al. 2008 (PMCID: PMC2556834 [72]), and finally HS seedlings from Schmid et al. 2005 (PMID:15806101 [33]), respectively. Ratios of expression between pollen and seedling are based on Loraine et al. 2013 (PMCID: PMC3668042 [22]). In cases where the seedling value was below the limit of detection, a minimal value of 0.0019 was substituted in its place as a denominator (0.0019 was the RPKM for ATCG00860 and was the lowest value reported in Loraine et al. 2013 (PMCID: PMC3668042 [22]). Ratio of expression between semi-in vivo pollen tube over dry pollen is based on Qin et al. 2009 (PMCID: PMC2726614 [23]). HS dependent changes in transcript abundance in shoots were based on publicly available data using the AtGenExpress Visualization Tool (AVT) (http://jsp.weigelworld.org/expviz/expviz.jsp, Schmid et al. 2005 (PMID:15806101 [33] for seedlings exposed to one hour HS at 38 °C). The log2-fold change was calculated based on a comparison of means of normalized values for two heat-stressed and two non-stressed seedling samples. NA stands for not available. Not Calculated, refers to a value not being calculated because one of the input sample read counts was considered to have an extreme outlier (see AT2G42540 and ATMG01360). (XLSX 8952 kb)

Additional file 4: Library size and principal component analysis. a. Table showing library sizes of each sample. b. A principal component analysis (PCA) of the filtered data showing that 87% of the variance of the samples can be explained by differences in the stress states. See methods for more details. Control and heat correspond to normal and HS conditions, respectively. (PPTX 43 kb)

Additional file 5: A transcript profile comparison to evaluate purity of pollen samples used for RNA-Seq. A subset of 12 genes was used to compare relative purities of pollen samples in the current pollen transcriptome study to those from a RNA-Seq study from Loraine et al. [22] (yellow highlights) or a microarray experiment from Qin et al. 2009 [23] (purple highlights). Four references genes were chosen to generate normalization factors that could be used to adjust expression values in Loraine et al. [22] and Qin et al. 2009 [23] to allow a relative comparison of the three data sets for WT pollen under control (normal) conditions. For a control group, three CNGC genes were chosen that displayed low to moderate levels of expression (Tunc-Ozdemir et al. 2013 [24] and Frietsch et al. 2007 [25]). As markers for potential contamination from photosynthetic tissues, five different nuclear encoded genes were chosen that are associated with either photosystems I/II, or chlorophyll A-B binding proteins (Umate 2010 [26]). Average relative ratios are shown for each of the four different pollen samples in comparison to both Loraine et al. [22] and Qin et al. [23]. (XLSX 19 kb)

Additional file 6: Integrated Genome Browser (IGB) screenshot showing *cngc16* RNA-Seq reads primarily upstream of *T-DNA* insertion site. The green arrow identifies the position of *T-DNA* insertion in *cngc16–2* (SAIL_726_B04). The observed reads aligning to *cngc16* are primarily on the 5′ side of the *T-DNA* disruption site, with only a few reads observed at two disconnected downstream positions. This suggests that there were no detectable full-length transcripts. (PPTX 66 kb)

Additional file 7: RNA-Seq validation using real-time Q-PCR. a. Comparison of expression values obtained from Q-PCR and RNA-Seq normalized to WT control (normal). The analysis was performed on two different reference genes separately (*CYCP2* (AT3G21870) and *UBQ7* (AT2G35635)). b. Primer sequences used for real-time Q-PCR. (XLSX 16 kb)

Additional file 8: Predicted targets for HS-modulated microRNAs. Target predictions for microRNAs were conducted with psRNATarget (http://plantgrn.noble.org/psRNATarget/, Dia and Zhao 2011 (PMCID: PMC3125753 [28])) using *Arabidopsis thaliana* unigene library (2017 update). *MIR156A* and *C* have the same targets. ND stands for not detected in current pollen transcriptome study. NA stands for not applicable because no target was predicted using psTarget. (XLSX 57 kb)

Additional file 9: Sorted lists of differentially expressed transcripts in WT and *cngc16* mutant. HS-dependent changes were sorted and extracted from the entire transcriptome analysis in Additional file 3. For genes listed in a given category, corresponding transcripts showed ≥2-fold change with an adjusted *p*-value ≤0.01. a. Tally of 21 pre-existing transcriptome differences identified in a direct comparison between WT and *cngc16* mutant pollen from unstressed (control/normal) plants. b. Tally of 471 HS-dependent transcriptome changes that were only classified as significant changes in WT. Genes shown include only WT-specific changes and do not include changes that were also observed to be in common with *cngc16* (see Additional file 9d). c. Tally of 2305 HS-dependent transcriptome changes that were only classified as significant changes in *cngc16*. Genes shown include only *cngc16*-specific changes and do not include changes that were also observed to be in common with WT (see Additional file 9d). d. Tally of 1631 HS-dependent transcriptome changes observed in both WT and *cngc16*. Genes shown include only common changes and do not include changes that were categorized as specific to WT or *cngc16* (see Additional file 9b and c, respectively). e. 192 transcriptome differences from a direct comparison between heat-stressed WT and *cngc16* mutant pollen. (XLSX 1638 kb)

Additional file 10: Association network for HS-dependent changes in WT that were most different in *cngc16* under HS. The figure was generated using STRING [32] and the top 23 genes identified in Table 3. (XLSX 154 kb)

Additional file 11: HS-dependent transcript abundance changes corresponding to transcription factors in WT and *cngc16* pollen. Differentially expressed transcription factors (TFs) are organized to show HS-dependent differences that are WT-specific (23 changes, yellow highlight), *cngc16*-specific (98 changes, blue highlight), or common to both mutant and WT (66 changes, white highlight). ^a^ A comparison is provided to HS-dependent changes observed for aerial parts of seedlings exposed to a one hour 38 °C HS based on publicly available microarray data in AtGenExpress, (http://jsp.weigelworld.org/expviz/expviz.jsp; Schmid et al. 2005 (PMID: 15806101 [34]). The log2-fold change was calculated based on means of normalized values for two heat-stressed seedlings compared to two non-stressed seedlings. NA stands for not available because of absence of probeset on microarray experiment. (XLSX 63 kb)

Additional file 12: 230 Genes with putative Ca^2+^-signaling related functions. 230 genes with putative Ca^2+^-signaling related functions were identified here with quantifiable expression levels in the pollen RNA-Seq data set. Of those 230, 40 genes (yellow highlights) showed HS-dependent changes (≥ 2-fold changes with adjusted *p*-value ≤0.01) in WT pollen. Similar HS-dependent changes were also observed for *cngc16* mutant, with seven potential exceptions with lower degrees of significance shown in bold. ^a^ Ratio of expression between pollen and seedling is based on Loraine et al. 2013 (PMCID: PMC3668042 [22]). ^b^ A comparison is provided to HS-dependent changes observed for aerial parts of seedlings exposed to a one hour 38 °C HS based on publicly available microarray data in AtGenExpress, (http://jsp.weigelworld.org/expviz/expviz.jsp; Schmid et al. 2005 (PMID: 15806101 [33]). The log2-fold change was calculated based on means of normalized values for two heat-stressed seedlings compared to two non-stressed seedlings. (XLSX 15 kb)

Additional file 13: A comparison of HS-dependent changes in pollen to 67 multi-stress response genes in vegetative tissues. From a list of 67 multi-stress genes curated by Swindell 2006 (PMCID: PMC1698639 [47]; highlighted in purple), 19 genes showed detectable expression in pollen. Among those, only three genes showed significant changes in pollen HS (red font). (XLSX 596 kb)

Additional file 14: GO analyses on HS-dependent changes in WT and *cngc16*. **a**. The number of genes in each GO category is shown for genes with HS-dependent changes observed in both WT (green header) and *cngc16* (orange header). Enrichment above an expected is shown along with a *p*-value. In a simple contrast analysis, a ratio of gene numbers in *cngc16* and WT is calculated for each GO category. The analysis was done as a PANTHER Overrepresentation Test (release 2017–04-13 [61]) using a GO Ontology database (released 2017–08-14) with 27,060 reference genes for *Arabidopsis thaliana*. NA stands for not applicable because no genes were detected in this category for either WT or *cngc16*. **b**. Uploads used for HS-dependent changes with ≥2-fold changes and adjusted *p*-value ≤0.01. (PPTX 138 kb)

Additional file 15: GO analysis on the 192 largest differences between WT and *cngc16* under HS. A GO analysis pie chart is shown for Molecular Function (a), Cellular Component (b), and Biological Process (c) generated using an upload of Additional file 3 or Additional file 9e column R listing the differences (≥ 2-fold and adjusted *p*-value ≤0.01) between WT and *cngc16* HS-transcriptomes. Categories were defined using PANTHER Overrepresentation Test (release 2017–04-13 [61]) using a GO Ontology database (released 2017–08-14) with 27,060 reference genes for *Arabidopsis thaliana*. Gene categories shown displayed enrichments with a *p*-value of ≤0.05. (PPTX 993 kb)

## Abbreviations

ACA: Autoinhibited Ca^2+^-ATPase
APX: Ascorbate peroxidase
ARF: Auxin Response Factor
Avg: Average
BAG6: BCL-2-associated athanogene 6
Ca^2+^: Calcium
CaLB: Calcium-dependent lipid-binding
CAM: Calmodulin
CAT: Catalase
CAX: Cation exchanger
CBL: Calcineurin B subunit-like protein
CBLD: Ca^2+^-dependent lipid-binding
CBN: Ca^2+^-binding endonuclease/exonuclease/phosphatase family
CHX: Cation/H^+^-exchanger
CIPK: Calcineurin B-like (CBL)-interacting protein kinase
CML: Calmodulin like
CNGC: Cyclic nucleotide-gated cation channel
cNMP: Cyclic-nucleotide monophosphate
Col-0 WT: Columbia-0 wild type
CPK: Ca^2+^-dependent protein kinases
CRT: Calreticulin
CYCP2;1: Cyclin p2;1
DELTA-VPE: Delta vacuolar processing enzyme
ER: Endoplasmic reticulum
Ez.: Enzyme
GO: Gene Ontology
HS: Heat stress
HSF: Heat shock transcription factor
HSP: Heat shock protein
HSR: Heat-stress response
IGB: Integrative Genome Browser
log2-CHG: Log2-fold change
LTP: Lipid transfer protein
Mbf1C: Multiprotein Bridging Factor 1c
MIR: MicroRNA
MS: Murashige and Skoog
Mut: cngc16
Norm: Normal
PCA: Principal component analysis
PE: Paired-end
Q-PCR: Quantitative PCR
ROS: Reactive oxygen species
Sig. adj. *p*-val: Significance based on adjusted *p*-value ≤0.01
SLP: Calcineurin-like metallo-phosphoesterase superfamily protein
sncRNA: Small non-coding RNA
SPL: Squamosa Promoter Binding Protein-Like
*T-DNA*: Transfer DNA
TF: Transcription factor
UBQ7: UBIQUITIN 7
UPR: Unfolded protein response
VAMP: Vesicle-associated membrane protein
WT: Wild type
