# Supplementary figures and images for: A comparison of heat-stress transcriptome changes between wild-type Arabidopsis pollen and a heat-sensitive mutant harboring a knockout of cyclic nucleotide-gated cation channel 16 (cngc16)
Source: BMC Genomics. 2018 Jul 24;19:549. doi: 10.1186/s12864-018-4930-4 (PMC6057101; doi:10.1186/s12864-018-4930-4)

## Slide 1
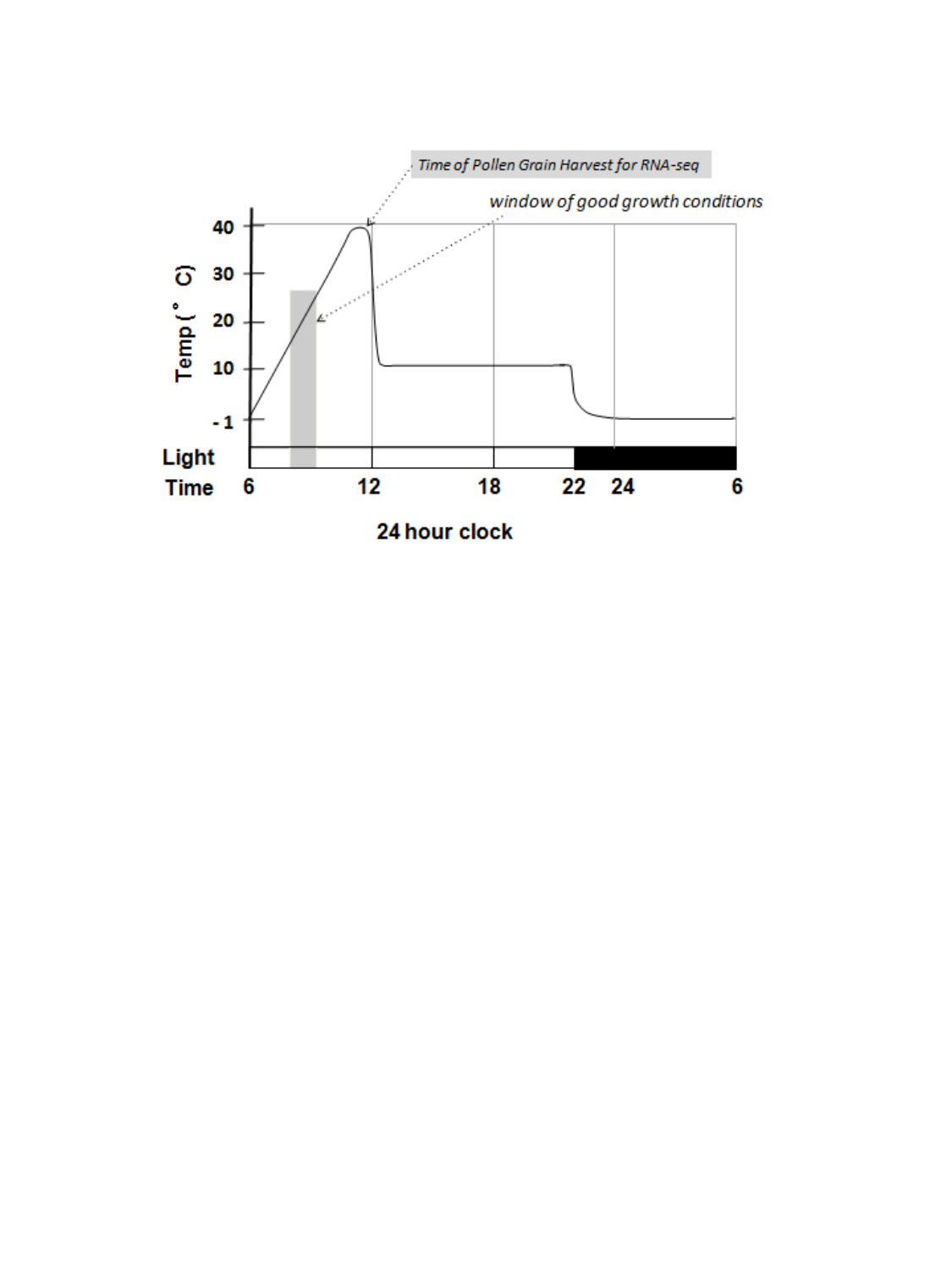

Supplement: Supplementary file 1 — Hot/Cold stress cycle. Diagram showing the Hot/Cold stress-cycle used here for growing plants from which pollen samples for RNA-Seq experiment were harvested at the end of HS-peak at 40 °C. See Methods and [20] for more details. (PPTX 50 kb) [file 12864_2018_4930_MOESM1_ESM.pptx]

## Slide 1
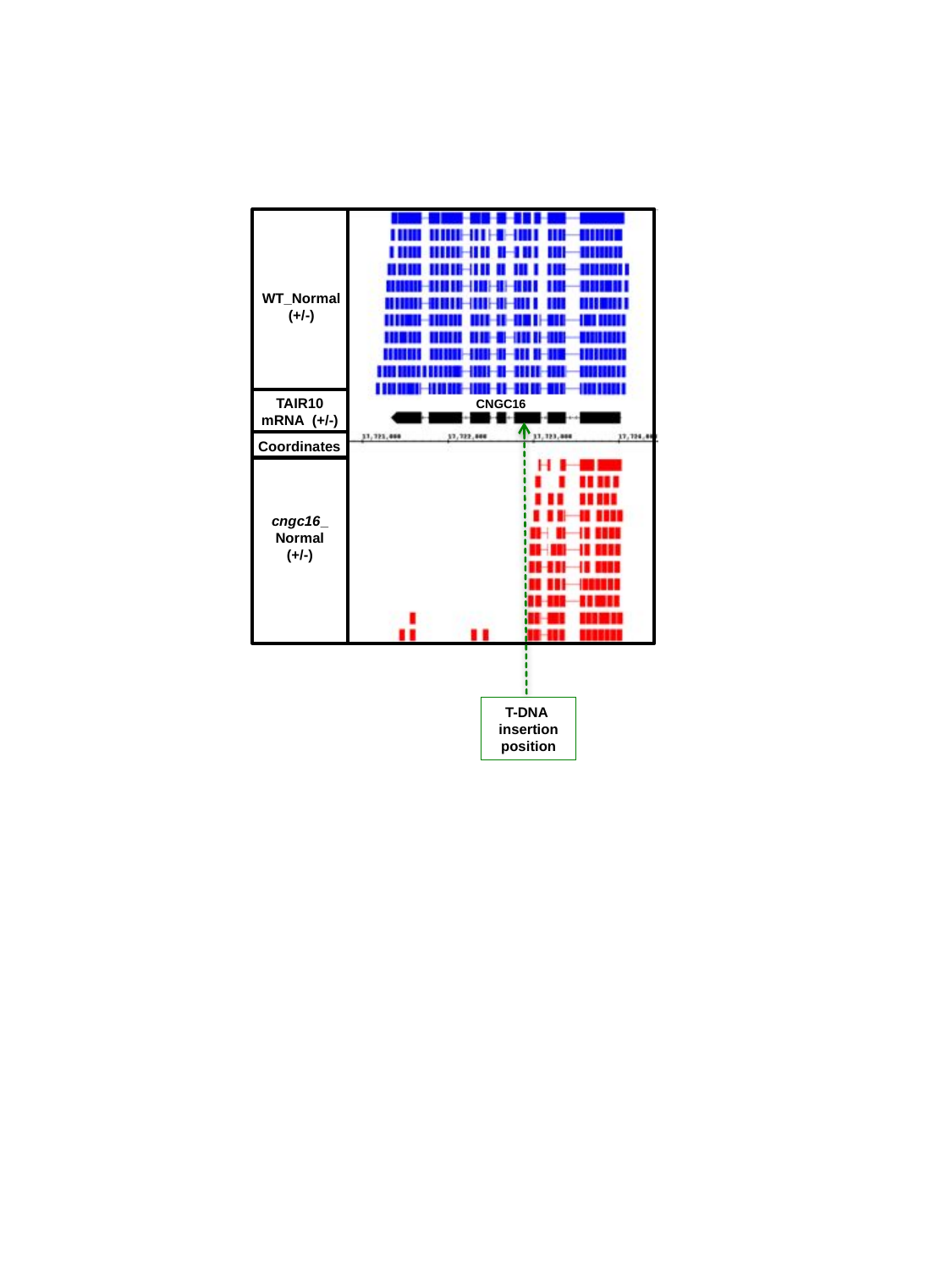

WT_Normal (+/-)
TAIR10
mRNA (+/-)
CNGC16
Coordinates
cngc16_
Normal (+/-)
T-DNA insertion position

Supplement: Supplementary file 6 — Integrated Genome Browser (IGB) screenshot showing cngc16 RNA-Seq reads primarily upstream of T-DNA insertion site. The green arrow identifies the position of T-DNA insertion in cngc16–2 (SAIL_726_B04). The observed reads aligning to cngc16 are primarily on the 5′ side of the T-DNA disruption site, with only a few reads observed at two disconnected downstream positions. This suggests that there were no detectable full-length transcripts. (PPTX 66 kb) [file 12864_2018_4930_MOESM6_ESM.pptx]

## Slide 1
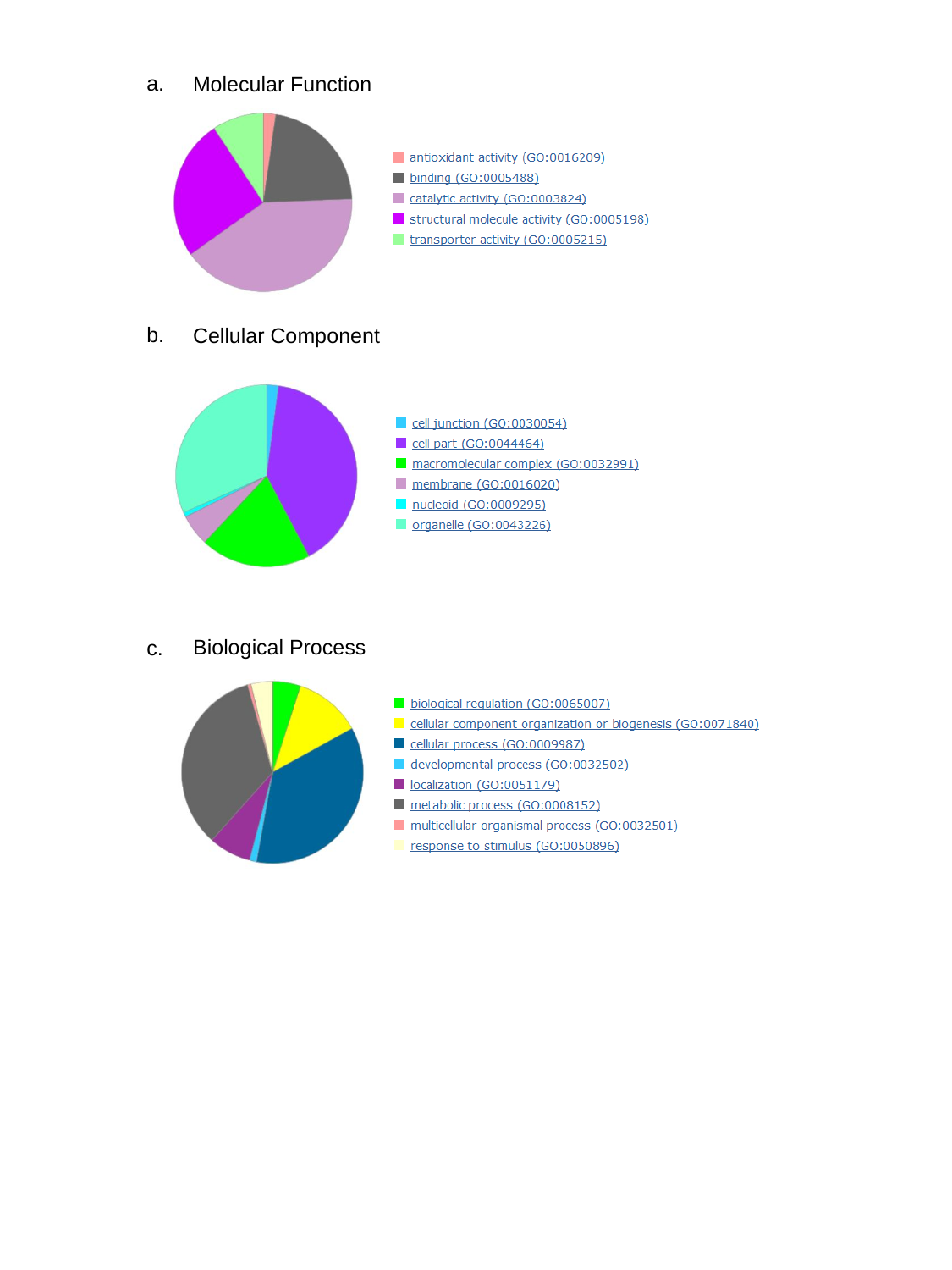

a.
Molecular Function
b.
Cellular Component
Biological Process
c.

Supplement: Supplementary file 14 — GO analyses on HS-dependent changes in WT and cngc16. a. The number of genes in each GO category is shown for genes with HS-dependent changes observed in both WT (green header) and cngc16 (orange header). Enrichment above an expected is shown along with a p-value. In a simple contrast analysis, a ratio of gene numbers in cngc16 and WT is calculated for each GO category. The analysis was done as a PANTHER Overrepresentation Test (release 2017–04-13 [61]) using a GO Ontology database (released 2017–08-14) with 27,060 reference genes for Arabidopsis thaliana. NA stands for not applicable because no genes were detected in this category for either WT or cngc16. b. Uploads used for HS-dependent changes with ≥2-fold changes and adjusted p-value ≤0.01. (PPTX 138 kb) [file 12864_2018_4930_MOESM14_ESM.pptx]

## Slide 1
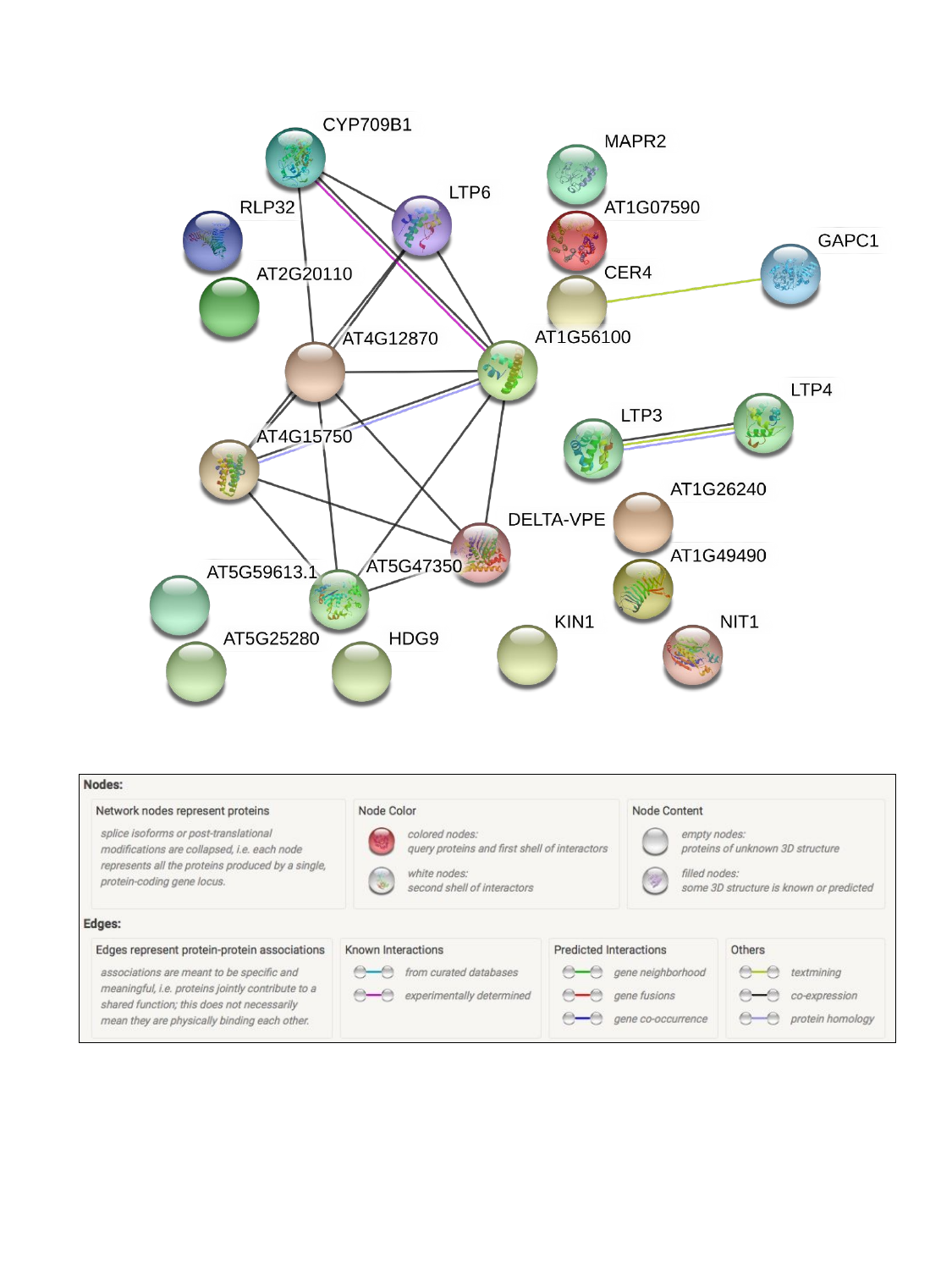

Supplement: Supplementary file 15 — GO analysis on the 192 largest differences between WT and cngc16 under HS. A GO analysis pie chart is shown for Molecular Function (a), Cellular Component (b), and Biological Process (c) generated using an upload of Additional file 3 or Additional file 9e column R listing the differences (≥ 2-fold and adjusted p-value ≤0.01) between WT and cngc16 HS-transcriptomes. Categories were defined using PANTHER Overrepresentation Test (release 2017–04-13 [61]) using a GO Ontology database (released 2017–08-14) with 27,060 reference genes for Arabidopsis thaliana. Gene categories shown displayed enrichments with a p-value of ≤0.05. (PPTX 993 kb) [file 12864_2018_4930_MOESM15_ESM.pptx]
