# Supplementary material for: A comparison of heat-stress transcriptome changes between wild-type Arabidopsis pollen and a heat-sensitive mutant harboring a knockout of cyclic nucleotide-gated cation channel 16 (cngc16)
Source: BMC Genomics. 2018 Jul 24;19:549. doi: 10.1186/s12864-018-4930-4 (PMC6057101; doi:10.1186/s12864-018-4930-4)
Supplement: Supplementary file 4 — Library size and principal component analysis. a. Table showing library sizes of each sample. b. A principal component analysis (PCA) of the filtered data showing that 87% of the variance of the samples can be explained by differences in the stress states. See methods for more details. Control and heat correspond to normal and HS conditions, respectively. (PPTX 43 kb) [file 12864_2018_4930_MOESM4_ESM.pptx]

## Slide 1
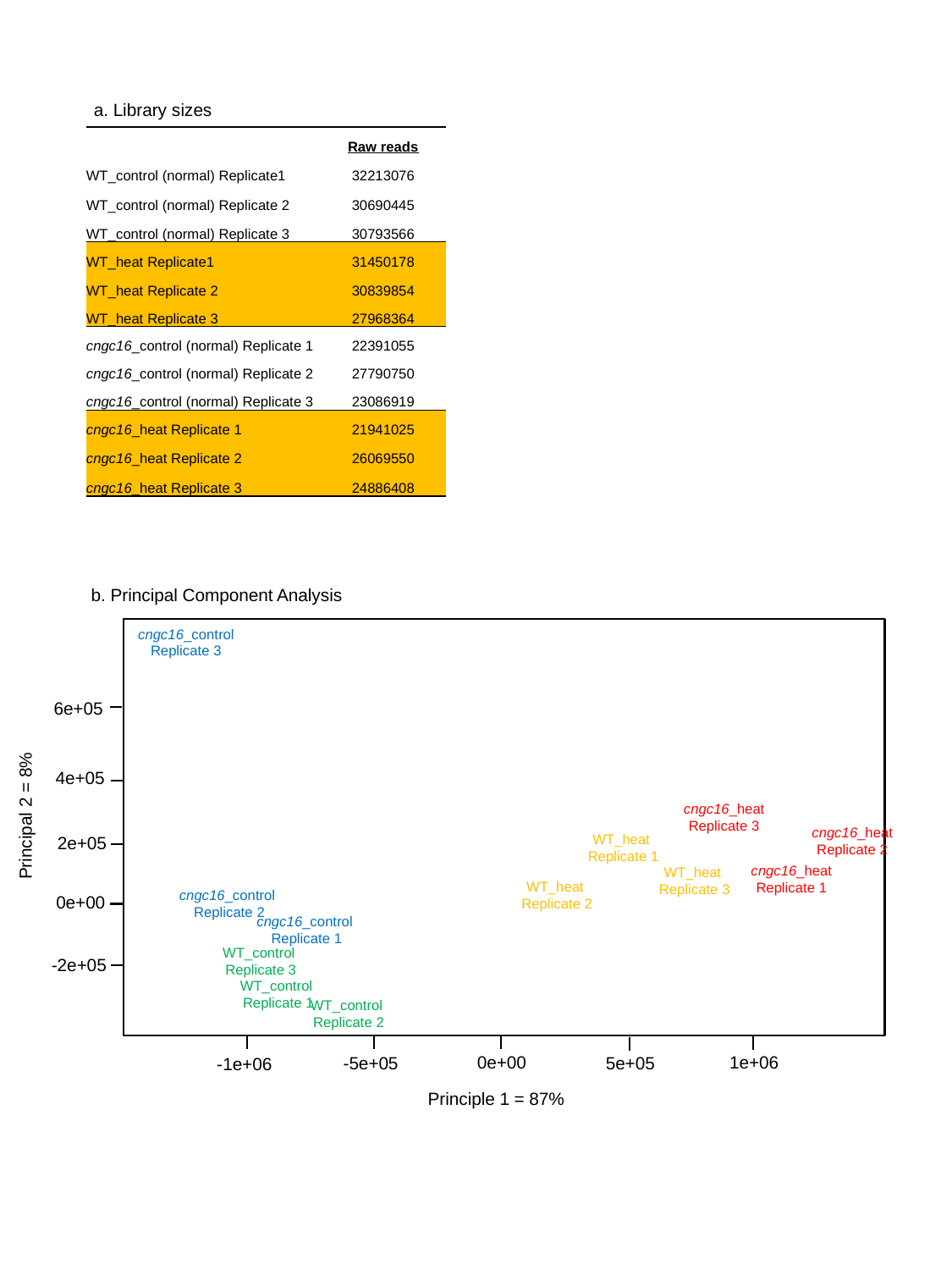

a. Library sizes
| | |
| --- | --- |
| | Raw reads |
| WT\_control (normal) Replicate1 | 32213076 |
| WT\_control (normal) Replicate 2 | 30690445 |
| WT\_control (normal) Replicate 3 | 30793566 |
| WT\_heat Replicate1 | 31450178 |
| WT\_heat Replicate 2 | 30839854 |
| WT\_heat Replicate 3 | 27968364 |
| cngc16\_control (normal) Replicate 1 | 22391055 |
| cngc16\_control (normal) Replicate 2 | 27790750 |
| cngc16\_control (normal) Replicate 3 | 23086919 |
| cngc16\_heat Replicate 1 | 21941025 |
| cngc16\_heat Replicate 2 | 26069550 |
| cngc16\_heat Replicate 3 | 24886408 |
b. Principal Component Analysis
cngc16_control Replicate 3
Principal 2 = 8%
cngc16_heat Replicate 3
cngc16_heat Replicate 2
WT_heat Replicate 1
2e+05
cngc16_heat Replicate 1
WT_heat Replicate 3
WT_heat Replicate 2
cngc16_control Replicate 2
0e+00
cngc16_control Replicate 1
WT_control Replicate 3
-2e+05
WT_control Replicate 1
WT_control Replicate 2
Principle 1 = 87%
6e+05
4e+05
1e+06
0e+00
5e+05
-5e+05
-1e+06
